# Origin of the Menagerie at the Jardin Des Plantes

**Published:** 1861-07

**Authors:** 


ORIGIN OF THE MENAGERIE AT THE JARDIN DES PLANTES.—Geoffroy St. Hilaire, at first a most laborious student at the Jardin des Plantes under the celebrated Fourcroy and Daubenton, exerted himself most heroically during the frightful massacres of September in rescuing the priests who had been his preceptors. Overcome with emotion, he then retired for awhile into the country, but, at the urgent solicitation of Hauy and Daubenton, he returned to Paris, and became one of the officials at the Jardin des Plantes. It was during the Reign of Terror, when the timid were only too glad to be replaced by more resolute friends; and first Daubenton, and then Lacepède, gave up the post of Demonstrator of the Cabinet of Natural History in favor of the courageous young Geoffroy. It was, indeed, to his bold and ingenious intervention and courageous fictions, which might have cost him his life, that these two great men owed their lives. Indeed, the proscribed of all parties found him their friend, converting as he did his apartments at the Jardin into a kind of asylum. By a strange contrast, amid all this confusion and bloodshed, a man of patriarchal simplicity and goodness of heart, Bernardin de St. Pierre, author of "Paul and Virginia," was placed at the head of the Jardin, now called the Museum of Natural History, with its twelve professors. A chair of zoology was created, but, being a new post, none of the professors would take it, and it fell to Geoffroy's lot as being the youngest, he being then only twenty-two. He had never hitherto taught anything but mineralogy; but he undertook his new task of teaching the history of the mammalia and birds with none the less resolution. The young professor, however, had but the fragments of a collection as objects of instruction; and as to a menagerie, France at that period knew of none other than that of the Gardens of Versailles. The simplest thing would have been to have transferred this to the Jardin, but during the period which followed August tenth the menagerie experienced the lot of everything attached to royalty, and underwent a true massacre. Most of the birds and quadrupeds were eaten, a rhinoceros and some lions, of which this use could not be made, having however been spared. These, too, were about to be sacrificed, when they found an eloquent and pathetic defender in the new intendant of the Jardin. The harmonious poet of nature, Bernardin de St. Pierre, constituted himself, in fact, their advocate, and pleaded their cause at the bar of the National Convention, taking as the epigraph to his discourse *Miseris succurrere disco*. More fortunate than others with the sentimental murderers who constituted the tribunal, he gained his cause. The lives of his clients were saved, and they were transferred to the Jardin des Plantes. Geoffroy added to them some ambulant menageries which the Commune of Paris had caused to be seized in the "cause of morality," and thus was laid the foundation of this valuable collection. — *Dubois' Eloge of Geoffroy St. Hilaire.*

